# AFRICAN IMMIGRANT BELIEFS AND CAREGIVER BURDEN: WHAT IS THE EVIDENCE?

**DOI:** 10.1093/geroni/igae098.0592

**Published:** 2024-12-31

**Authors:** Manka Nkimbeng, Truphosa Aswani, Elizabeth Albers, Joseph Gaugler

**Affiliations:** University of Minnesota, Minneapolis, Minnesota, United States; University of Minnesota, Minneapolis, Minnesota, United States; University of Minnesota, Minneapolis, Minnesota, United States; University of Minnesota, Minneapolis, Minnesota, United States

## Abstract

Perceptions among African immigrants can impact caregiving for individuals with dementia and their caregivers. However, the specific beliefs and their effects on caregiving outcomes remain unclear. This study aims to investigate the relationship between African immigrants’ beliefs and caregiver burden. Logistic regressions were used to explore the relationship between various beliefs (including family homecare, secretiveness about health, fear of bad news and death, facility-level care being the last resort, and preference to send someone with dementia to Africa) with caregiver burden with 155 African immigrant caregivers. The average age of a caregiver was 34 years, 30% were born in Africa, and 12% of persons living with dementia were residing in Africa. After adjusting for age, sex, place of birth, and marital status, caregivers who believed that care should be provided at home, those who viewed facility-level care as a last resort, and those who preferred to send someone with dementia to Africa showed higher odds of caregiver burden, although these associations were not statistically significant (OR 1.19, 95% CI [0.42-3.4]; OR 1.16, 95% CI [0.42-3.18]; OR 1.09, 95% CI [0.37-3.24], respectively). Although these findings were not statistically significant, the trend reveals that these African immigrant beliefs may negatively influence the care partner. Thus, there is a need for interventions that target both beliefs and poor health outcomes in Africans, an understudied research population.

